# Starting to disentangle the taxonomy of *Neocordulia* Selys (Odonata: Idomacromiidae): a review of the Atlantic Forest dragonfly *N. mambucabensis* Costa & T.C. Santos

**DOI:** 10.7717/peerj.20991

**Published:** 2026-05-13

**Authors:** Juliana Ehlert, Jessica L. Ware, Ângelo Parise Pinto

**Affiliations:** 1 Graduate Program in Ciências Biológicas (Entomologia)—PPGEnto, Departamento de Zoologia, Universidade Federal do Paraná, Curitiba, Paraná, Brazil; 2 Laboratory of Systematics on Aquatic Insects (LABSIA), Departamento de Zoologia, Universidade Federal do Paraná, Curitiba, Paraná, Brazil; 3 Invertebrate Zoology, American Museum of Natural History, New York, NY, United States of America

**Keywords:** Anisoptera, Endemic, Species delimitation, Larvae morphology, Aquatic insects

## Abstract

The anisopteran *Neocordulia* Selys, 1882 is the most species-rich genus among all Corduliidae *s.l.* in the Neotropical region, and its taxonomy is plagued with inconsistencies, misconceptions and weak species-level hypotheses. This is mostly due to the boost in the number of species described since the most recent revision in the 1990s. Many species are known only from a single specimen that emerged in laboratory conditions, reared from larvae, and many are pending revision. This genus is a key group for understanding the phylogeny and spatial and morphological evolution of Corduliidae *s.l.* as part of Idomacromiidae, a clade including four other genera: *Idomacromia* Karsch, 1896, *Nesocordulia* McLachlan, 1882, *Oxygastra* Selys, 1870 and *Syncordulia* Selys, 1882. *Neocordulia mambucabensis* Costa & T.C. Santos, 2000, a Brazilian Atlantic Forest endemic dragonfly, is one of the poorly known species of the genus. The morphology and taxonomic status of adults and larvae of this South American emerald are reviewed based on the study of specimens from the southernmost known population for this species and compared to the type series. Illustrations, including images from the types, collection and alive specimens, as well as details of its habitat are provided. The male is rediagnosed, while our results do not support the female “allotype” as belonging to *N. mambucabensis*, so it is redescribed based on unequivocal specimens. The morphology of ultimate stadium larvae (F-0) is described, diagnosed, and illustrated for the first time. Because of numerous inaccuracies in literature, resulting in deficient knowledge of the taxonomy of *Neocordulia*, the genus requires further revision. Based on the information currently available, it is not possible to definitively provide adequate diagnoses for *Neocordulia* species.

## Introduction

As many other corduliids once included in the heterogenous assemblage Gomphomacromiinae of Tillyard & Fraser (1940), a taxon later recovered as polyphyletic (*e.g.*, Ware, May & Kjer, 2007; Bybee et al., 2021; Newton et al., 2026), the 19th century *Neocordulia* Selys, 1882 (Selys-Longchamps, 1882) has presented a challenging taxonomic, phylogenetic, and evolutionary history. Considered as one of the most important groups for understanding the evolution of Libelluloidea lineages, *Neocordulia* s.l. is the most species-rich genus among all genera formerly known as corduliids in the Neotropical region, comprising 17 species (see Paulson et al., 2026 for the number of species). Most of its richness stems from the profusion of descriptions between 2000 and 2010, when eight new species were introduced, doubling to the current number of known species after the revision published more than three decades ago by May (1992).

The genus’ taxonomic history started with the four Selysian species originally included in the Andean and enigmatic *Gomphomacromia* Brauer, 1864 (Selys, 1871, 1874) that were further grouped into the newly erected *Neocordulia* Selys, 1882. Since the genus revision (May, 1992), two distinct subgenera from morphological and biogeographical standpoints have been recognized, the *Mesocordulia* May, 1992, with four species ranging from south of North America in Mexico (González-Soriano, 1985), throughout Central America (May, 1992) to northwestern of South America (Pinto & Carvalho, 2011), and *Neocordulia* s.s. with 13 species, endemic to South America (Pinto & Carvalho, 2011; De Marmels, 2024). Within this latter subgenus, suggestions were made about species relationships (May, 1992; Machado, 2005; Costa & Machado, 2007) and two groups had been informally recognized based on morphology of adult male cercus: (1) the *Neocordulia androgynis*-group, composed by *N. androgynis* Selys, 1871, *N. biancoi* Rácenis, 1970, *N. carlochagasi* Santos, 1967, *N. fiorentini* Costa & Machado, 2007, *N. santacatarinensis* Costa et al. 2008; and (2) the *Neocordulia setifera*-group, with *N. gaucha* Costa & Machado, 2007, *N. machadoi* T.C. Santos et al., 2010, *N. mambucabensis* Costa & T.C. Santos, 2000, *N. matutuensis* Machado, 2005, *N. pedroi* Costa, Carrico & T.C. Santos, 2010, *N. setifera* (Hagen *in* Selys, 1871) and *N. volxemi* Selys, 1874. The most recent addition to the genus, the Venezuelan *Neocordulia maurocostai* De Marmels, 2024, was not placed into any of these species’ groups (De Marmels, 2024).

Due to their diversity and widespread distribution, the taxonomy of *Neocordulia* is plagued by imprecisions, misidentifications, and taxonomic mistakes (Araujo & Pinto, 2021). Until just recently, the genus had never been included in phylogenetic studies based on molecular data, being considered an *incertae sedis* taxon in terms of its relationship to other Corduliidae *s.l.* (see Dijkstra et al., 2013; Bybee et al., 2021). In the past, *Neocordulia* was included in the family Oxygastridae Bechly, 1996 based on intuitive inference of morphological data, together with the Australian *Hesperocordulia* Tillyard, 1911 and the European *Oxygastra* Selys, 1870 (Bechly, 1996), a genus to which *Neocordulia* has been even considered a junior synonym (Fleck, 2018). Oxygastridae was used inconsistently in literature with different sets of genera (*e.g.*, Bechly, 1996; Theischinger & Hawking, 2006; Fleck, 2018). The most recent phylogenetic propositions based on genomic data using a large dataset through anchored hybrid enrichment (AHE) and morphological characters (Goodman et al., 2026; Newton et al., 2026) solved most of these classification inconsistencies. In these analyses, *Neocordulia* was included in a well-supported clade together with the African *Idomacromia* Karsch, 1896, and *Syncordulia* Selys, 1882, the Malagasy *Nesocordulia* McLachlan, 1882 and the European *Oxygastra*, into the new family status of Idomacromiidae Tillyard & Fraser, 1940 (for details see Goodman et al., 2026), which may imply an old biogeographical evolution. Shortly before, De Marmels (2024) suggested elevating the subgenus *Mesocordulia* to the status of a full genus, thus *Neocordulia* s.s. would be represented exclusively by the 13 South American species. All these hypotheses are pending robust phylogenetic analyses at genus and species-levels and since the limits of *Neocordulia* species are not well defined, the relationships between them are also unclear. This knowledge void becomes evident when considering that most of the species of *Neocordulia* are known by a single holotype or limited to a few specimens from their type series.

*Neocordulia mambucabensis* was described based on a male and a female reared from larvae and emerged in laboratory. They were collected during one of the expeditions of the renowned Brazilian odonatologist, Newton Dias dos Santos, to the Rio Mambucaba River in the 1970′s, located about of 1,500 m of elevation at Serra da Bocaina National Park (PNSB), southeastern Brazil (Costa & Santos, 2000b). Thirty-five years later, a second population was reported in one of the richest global sites for odonates in the Serra dos Órgãos mountain range, at an elevation of 1,050 m, in the state of Rio de Janeiro (Kompier, 2015). Both series of specimens from N. D. Santos and T. Kompier were deposited in the Museu Nacional of Federal University of Rio de Janeiro and lost in the fire that destroyed most of the museum collections, hence no specimen of *N. mambucabensis* remained in natural history collections. Back in 2018, a third population was discovered in southern Brazil at 1,000 m of elevation in Mananciais da Serra Protected Area (MASE), within the Pico do Marumbi State Park, Paraná State (Araujo & Pinto, 2021).

The study by Araujo & Pinto (2021) is the most recent contribution to the knowledge of this species. They observed substantial differences between the female described by Costa & Santos (2000b) and photographs of the male holotype, leading to a hypothesis that the female paratype may not belong to *N. mambucabensis*. Coupled with the reported differences, Araujo & Pinto (2021) questioning is reinforced by the fact that at least two other *Neocordulia* species are sympatric and occur in the same area of Serra da Bocaina (*N. androgynis* and *N. setifera*, see Costa et al., 2000), implying that the paratype female may belong to another species occurring at the same location and could have been misidentified. In addition, the distinction between *Neocordulia* females can be confusing due to similarities or absence of exclusive characters. This is aggravated by their limited representation in collections, which results in the intraspecific phenotypic variations not being well understood. Further highlighting the need for more information about species identities, larvae are known for only slightly more than half of the species, mostly described based on a single exuvia or associated with adults by questionable evidence, such as locality only. Also, the diagnostic characters selected for identification keys of larvae have been often misinterpreted or reflect sexual characteristics, such as the proportions between caudal appendages’ length.

As part of an ongoing taxonomic revision of the whole genus *Neocordulia* s.l., the goal of this study is to elucidate the identity of *N. mambucabensis* and provide new descriptions for both males and females, with illustrations, diagnoses, updated records and the first description of its larvae.

## Materials & Methods

### Specimen repositories

Type material held in the Museu Nacional was lost, but it was examined by APP and during herein study through photographs taken in 2012, before the fire. Specimens examined are deposited in the following institutions:

ABMM—Coleção Especial Angelo Barbosa Monteiro Machado, Taxonomic Collections Center (CCT-UFMG), Department of Zoology, Universidade Federal de Minas Gerais, Belo Horizonte, MG, Brazil;
DZRJ—Entomological Collection Coleção Entomológica Prof. José Alfredo Pinheiro Dutra, Departamento de Zoologia, Instituto de Biologia, UFRJ, Rio de Janeiro, RJ, Brazil;
DZUP—Entomological Collection Pe. Jesus Santiago Moure, Departamento de Zoologia, Universidade Federal do Paraná, Curitiba, PR, Brazil http://grbio.org/cool/5xp9-edpx;
MNRJ—Entomological Collection, Departamento de Entomologia, Museu Nacional, Universidade Federal do Rio de Janeiro, Rio de Janeiro, RJ, Brazil https://doi.org/10.15468/7lklen;
RBINS—Royal Belgian Institute of Natural Sciences, Brussels, Belgium http://grbio.org/cool/c7r2-61q8.

### Collecting expeditions and permits

Field expeditions were conducted to the type locality (PNSB) and MASE during 2022–2024. Adults were captured with entomological aerial nets and Malaise traps. Larvae were collected with the help of sieves; some specimens were immediately fixed and preserved in absolute ethanol (100%) for molecular studies and others were kept alive to be reared in the laboratory until the emergence of adults. Laboratory maintenance of the larvae followed techniques detailed by Carvalho (2007), with specimens being kept in individual containers and monitored regularly till adult emergence. Samples acquisitions were conducted under collecting licenses and permits provided by Companhia de Saneamento do Paraná–SANEPAR, Instituto Chico Mendes de Conservação da Biodiversidade–ICMBIO/SISBIO (#59681) and Instituto Água e Terra–IAT (04.18, 037.2018, 03.20 and 52.23).

### Morphological analysis and species identification

The catalog list was elaborated consulting all references from 2000 to 2025 that mentioned the keywords *Neocordulia* or the specific name *mambucabensis*, such as original description, checklists, catalogs, and online databases (*e.g.*, Web of Science). Specimens were examined with the aid of a stereomicroscope or photos. To aid our morphological comparative analysis, species included in *N. setifera*-like group were also examined: *N. machadoi*, *N. mambucabensis, N. matutuensis, N. setifera* and *N. volxemi*. We also examined photographs from the type series, as well as all material previously studied and determined as *N. mambucabensis* by Araujo & Pinto (2021) which most emerged in laboratory. Additional larvae specimens from MASE were compared and associated with this species based on the larval exuviae of emerged adults. The specimens collected at type locality, the Rio Mambucaba River, were compared with exuviae and larvae from MASE and to one female that had partly emerged; these comparisons were used to associate this population with *N. mambucabensis*. Measurements were obtained with a micrometer eye piece attached to a stereomicroscope at 12x, 16x, 25x, and 32x, following standard measurements (*e.g.*, body and wing lengths) and others specific dimensions adopted to *Neocordulia* in the original description and genus revision (*e.g.*, May, 1992). Only well-preserved specimens were used to determine body and caudal appendages size, to avoid biases arising from incompletely expanded specimens, malformation after emergence or post-mortem distortions.

### Terminology

General morphological structures follow the terminology adopted in recent articles on Neotropical corduliids (*e.g.*, Ehlert & Pinto, 2020; Pinto, Almeida & Ehlert, 2022). Wing venation terminology follows Riek & Kukalová-Peck (1984) with additions summarized in Pinto (2024). For the male vesica spermalis (VS), the nomenclature proposed by Pfau (2011) was followed. The female terminalia nomenclature used is based on Matushkina (2011) and the external genitalia referred to here is the subgenital plate (lobes) and distally projected parts of the S8 sternite. Larval general morphology terminology follows Snodgrass (1954) and Carvalho & Kloosterman (2001), the labium and mandible terminology follows Corbet (1953) and Watson (1956), respectively. Family-level classification follows Goodman et al. (2026, First published on 2025, date used for nomenclatural purposes), with *Neocordulia* included within *Idomacromiidae* Tillyard & Fraser, 1940 (Tillyard & Fraser, 1940). The following abbreviations were used in the text: a.s.l., above sea level; Ax, antenodal crossvein; Fw, fore wing; Hw, hind wing; pt, pterostigma; Px, postnodal crossvein; S1–10, abdominal segments 1 to 10; VS, vesica spermalis; V1–4, segments of vesica spermalis.

### Geodata and images

Distributional and elevational records from the literature and specimen labels were compiled into a digital database. Coordinates that were missing from specimen labels or the original publications were taken from online gazetteers such as IBGE (2011) and eventually adjusted in Google Earth Pro (https://www.google.com.br/earth/download/gep/agree.html). A map was produced using the freeware QGIS 3.36. Images of specimens were obtained with a DSLR camera attached to a motorized rail system by MJKZZ using Helicon Remote (4.4.3 W) and source images stacked with the aid of Helicon Focus (*v.* 8.8.2, https://www.heliconsoft.com/). Type specimens and labels were scanned in HP scan during 2012 by APP.

## Results

### Species delimitation and recognition

**Figure 1:**
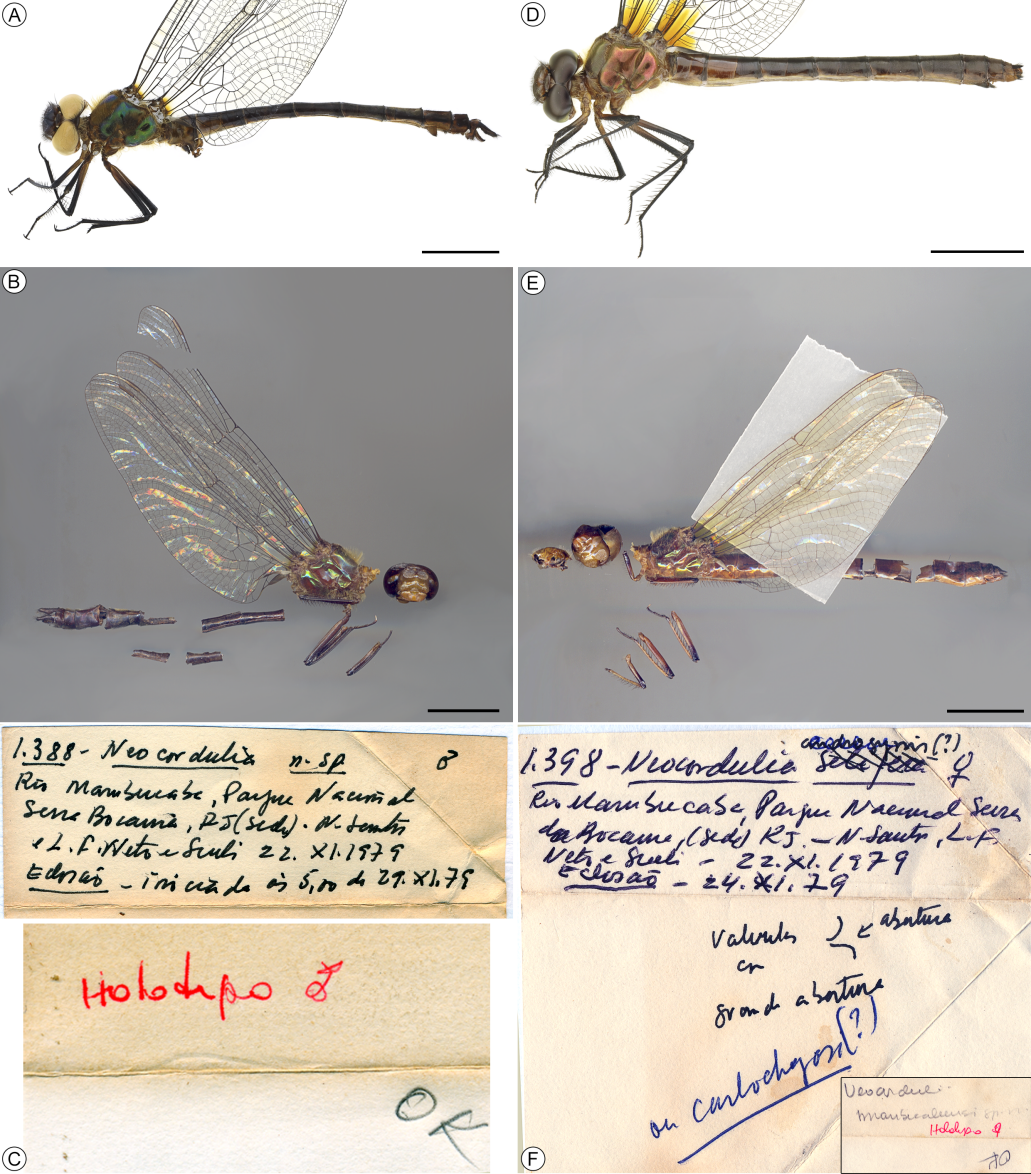
Habitus and labels of specimens of *Neocordulia mambucabensis*. Males (A–C) and females (D–F): (A) Specimen from Paraná state (DZUP); (B) holotype and (C) corresponding labels from Rio Mambucaba (MNRJ); (D) Specimen from Paraná state (DZUP); (E) allotype and (F) corresponding labels (MNRJ); Both type specimens lost in the fire. Scale bars = 10 mm.

**Figure 2:**
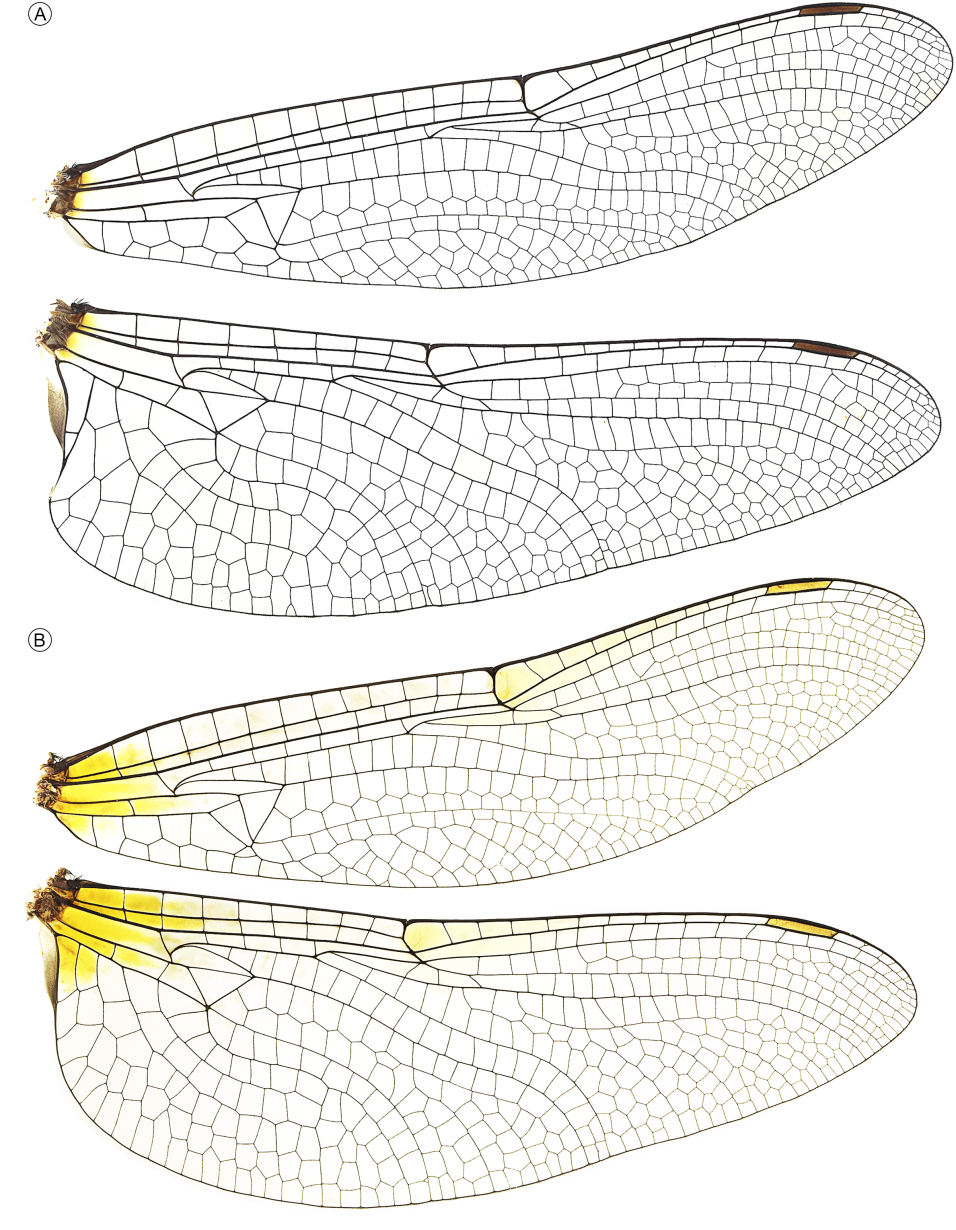
Wings of male (A) and female (B) of *Neocordulia mambucabensis* from Paraná, Brazil (DZUP). Among all examined specimens, only the female forewing in B. possesses a single bridge crossvein.

**Figure 3:**
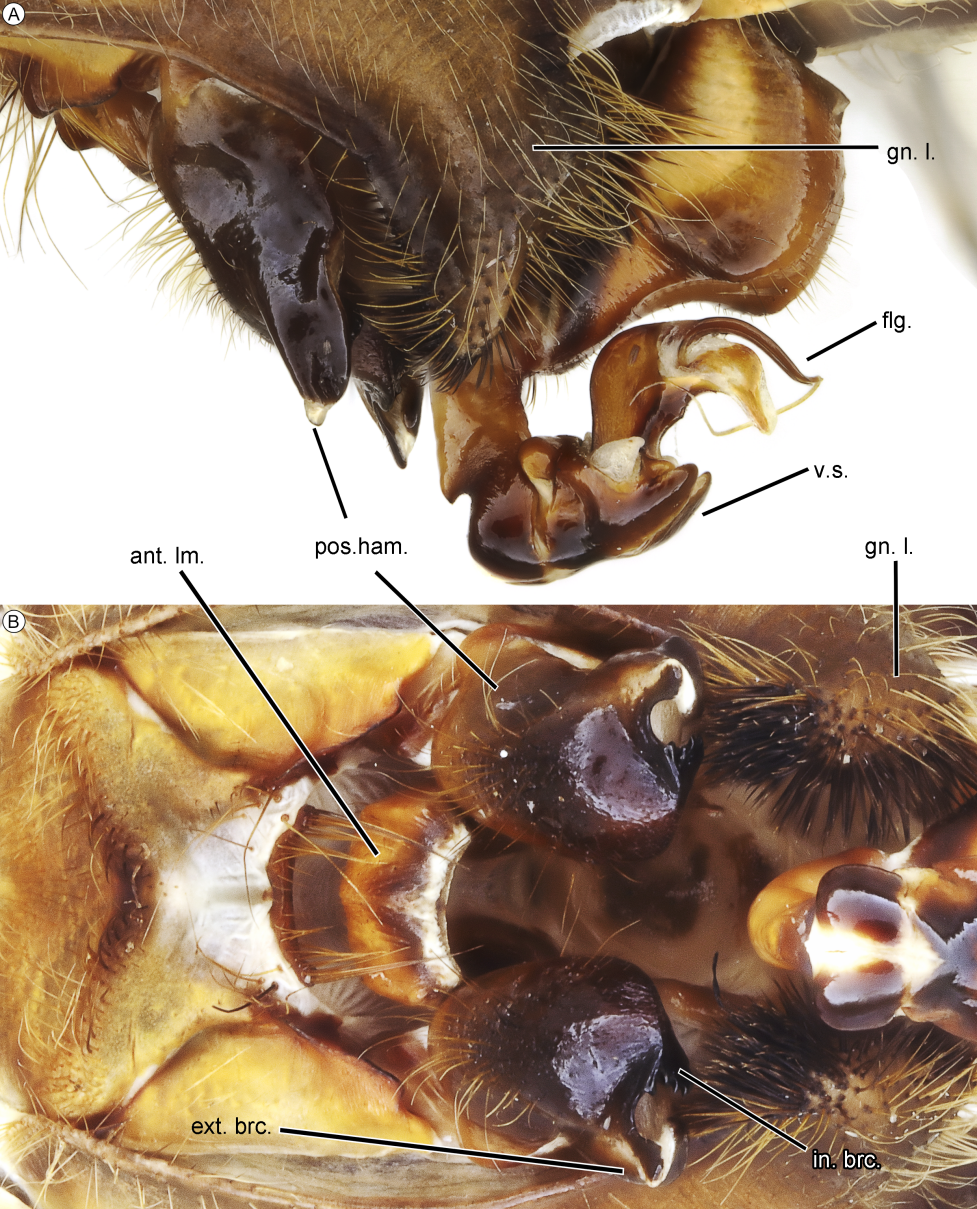
Secondary genitalia of male of *Neocordulia mambucabensis* from state of Paraná, Brazil (DZUP) in lateral (A) and ventral (B) views. Abbreviations: an. lm., anterior lamina; ext. brc., external branch; flg., flagellum; gn.l, genital lobe; in. brc., inner branch; pos. ham., posterior hamule; v.s., vesica spermalis.

**Figure 4:**
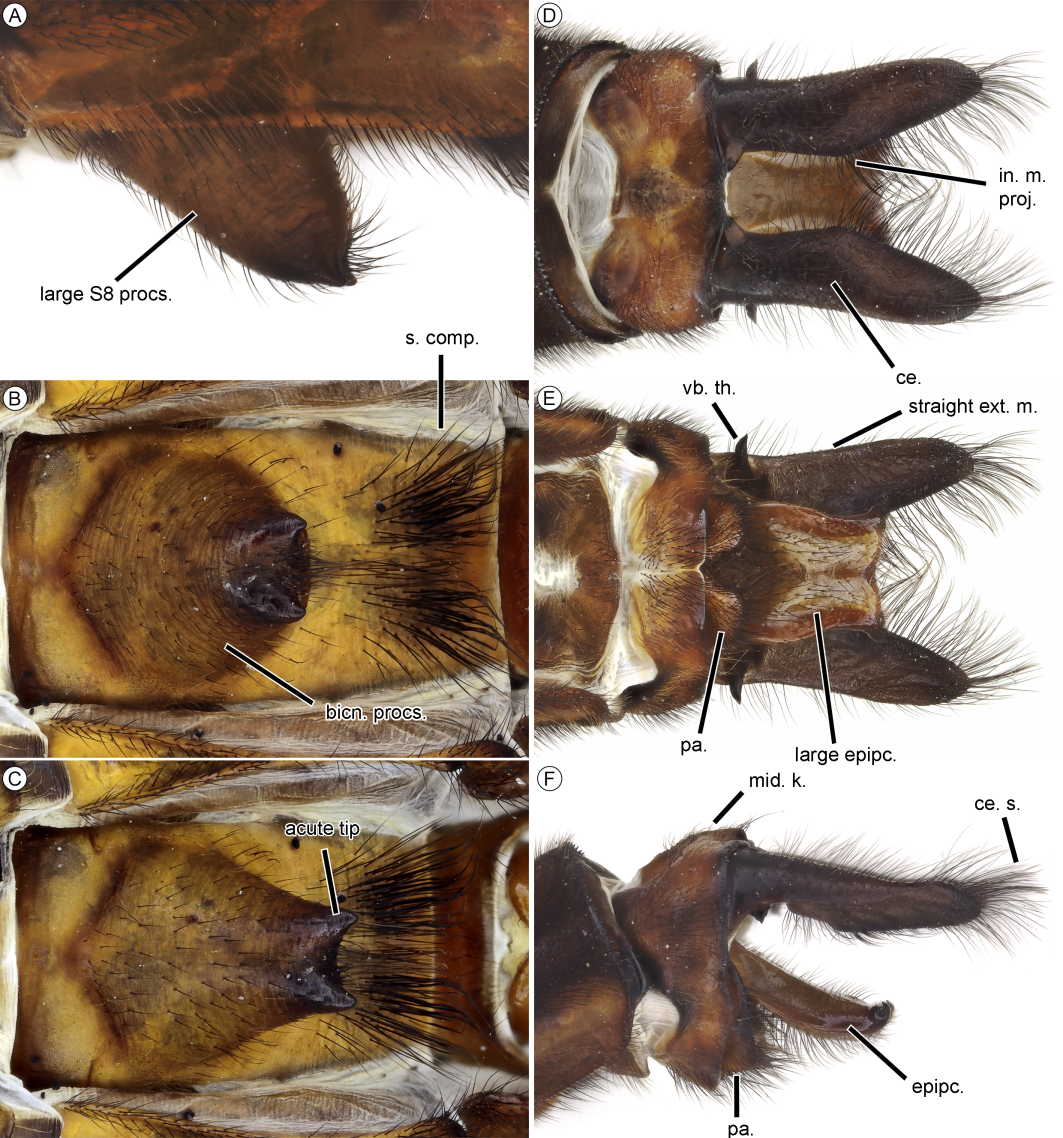
Male of *Neocordulia mambucabensis* from Paraná state, Brazil (DZUP). (A–C) Sternal process S8 in lateral, ventral and anteroventral views; (D–F) caudal appendages in dorsal, ventral and lateral view. Abbreviations: bicn. procs., biconical process; ce., cercus; ce. s., cercus setae; epipc., epiproct; ext. m., external margin; in. m. proj., inner margin projection; mid. k., middorsal keel; pa., paraproct; s. compl., setae complex; vb. th., ventrobasal tooth.

**Figure 5:**
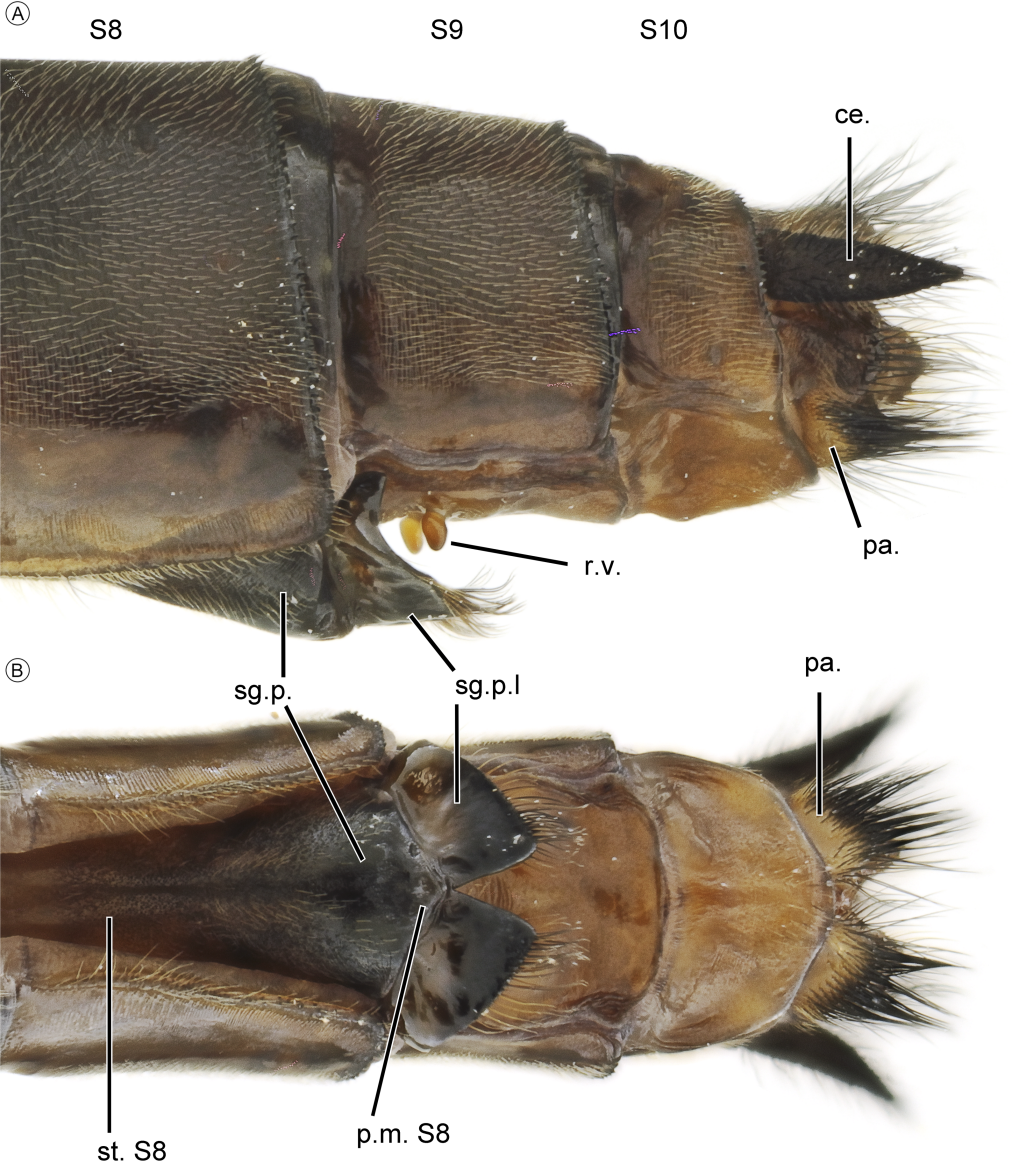
Female of *Neocordulia mambucabensis* from Paraná state, Brazil (DZUP). (A) caudal appendages in lateral view; (B) subgenital plate in ventral view. Abbreviations: ce., cercus; pa., paraprocto; p.m. S8, posterior margin of segment 8; r. *v.*, rudiments of valvae; sg.p., subgenital plate; sg.p.l, subgenital plate lobe; st. S8, sternite of segment 8.

**Figure 6:**
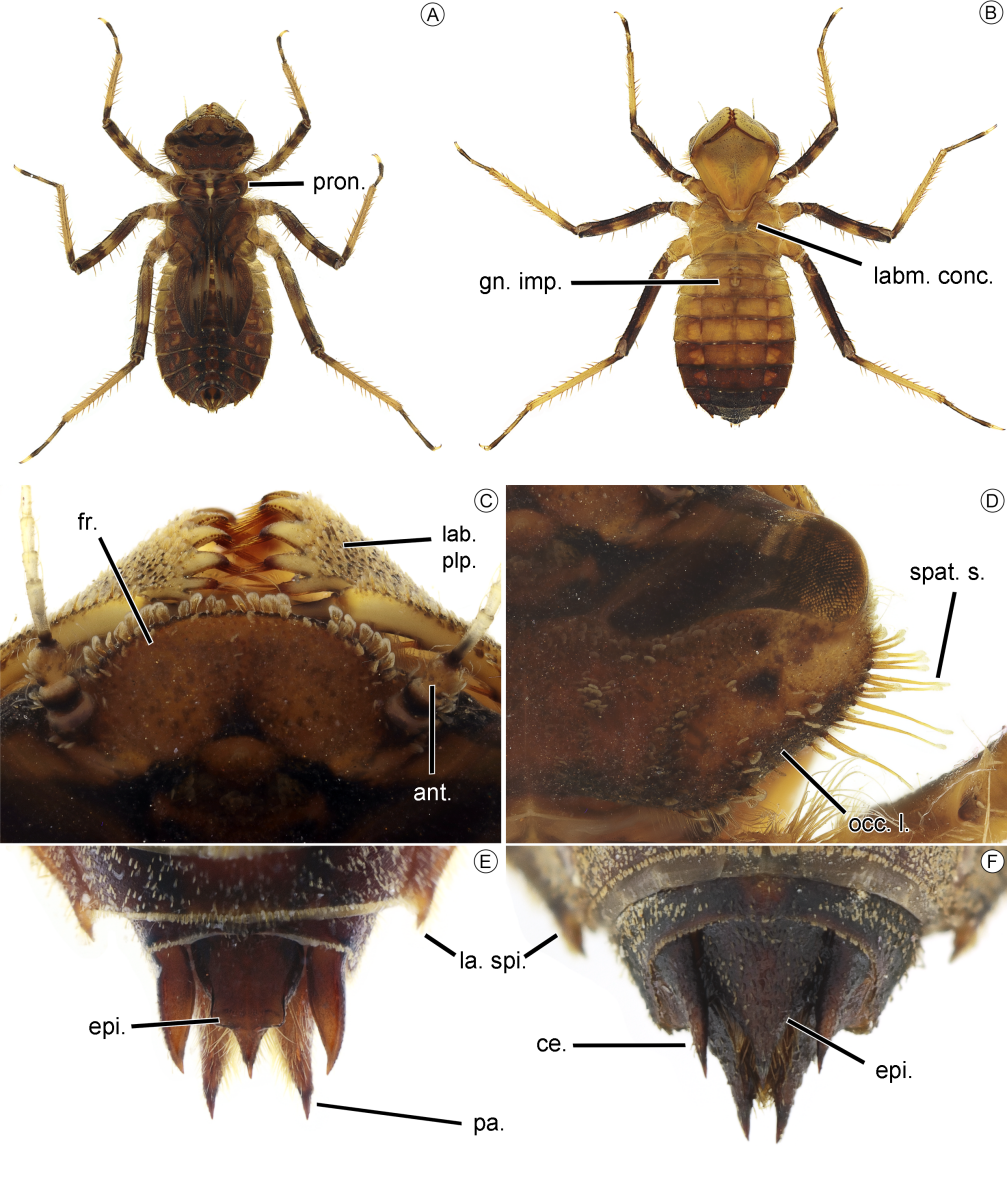
Larvae of *Neocordulia mambucabensis*. F-0 male larva from São Paulo state, Brazil (A–E) and female exuvia from Paraná state, Brazil (F) of *Neocordulia mambucabensis* (DZUP): (A–B) habitus in dorsal and ventral views; (C) frontal plate in dorsal view; (D) occipital angle; (E–F) anal appendages in dorsal view. Abbreviations: ant., antenna; ce., cercus; epi., epiprocto; fr., frons; gn. imp., genital impression; lab. plp., labial palp; labm. conc., labium concavity; occ. l., occipital lobe; sp., spines; pa., paraprocto; pron., pronotum.

**Figure 7:**
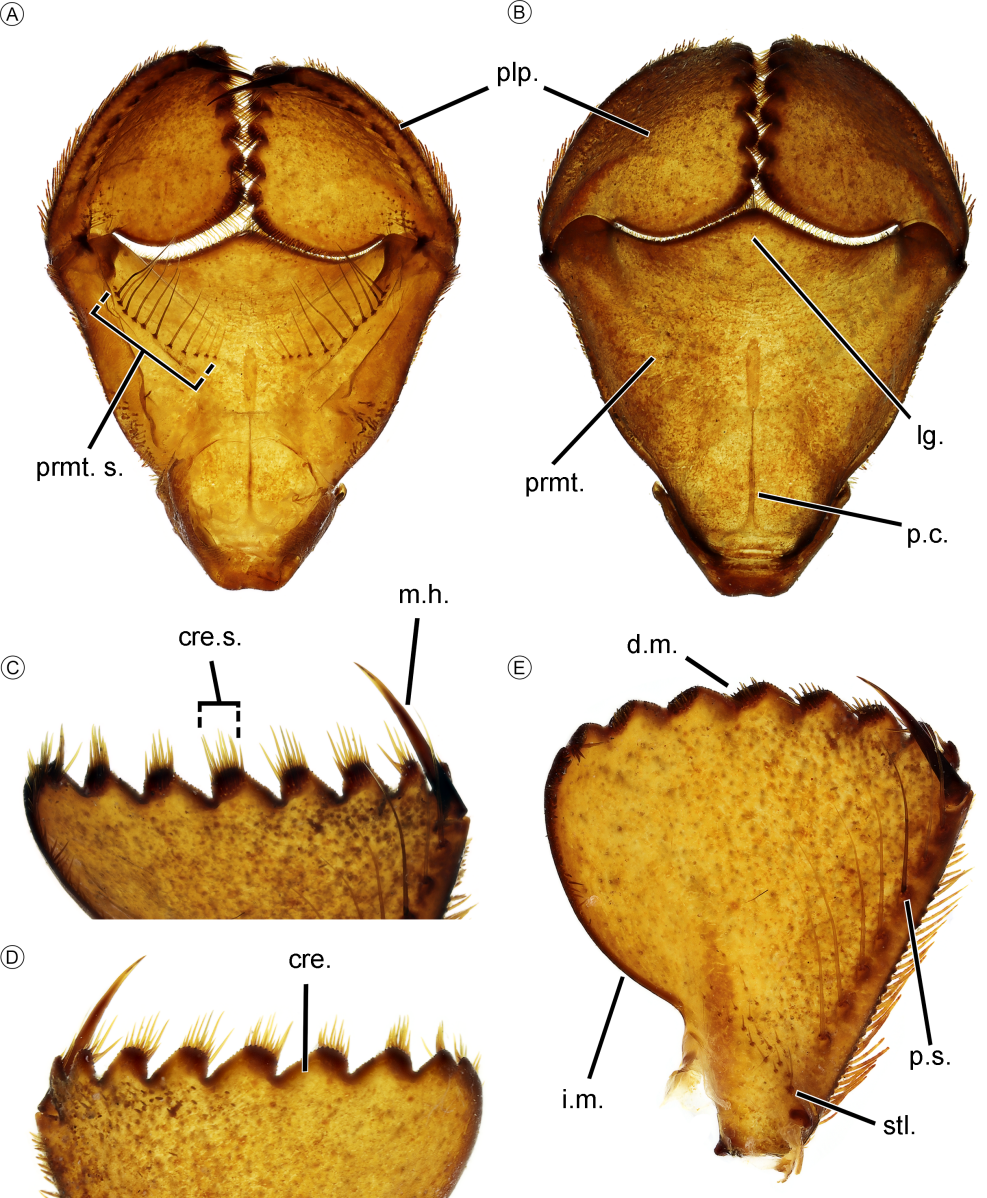
Mouth parts of *Neocordulia mambucabensis* larva from Paraná state, Brazil (DZUP). (A–B) prementum in dorsal and ventral views; (C–D) labial palps crenulations in dorsal and ventral views; (E) labial palp in dorsal view. Abbreviations: cre., crenulation; cre. s., crenulation setae; d. m., distal margin; i. m., inner margin; lg., ligula; m. h., movable hook; plp., palp; p. s., palpal setae; prmt., prementum; prmt. s., prementum setae; stl., setella.

**Figure 8:**
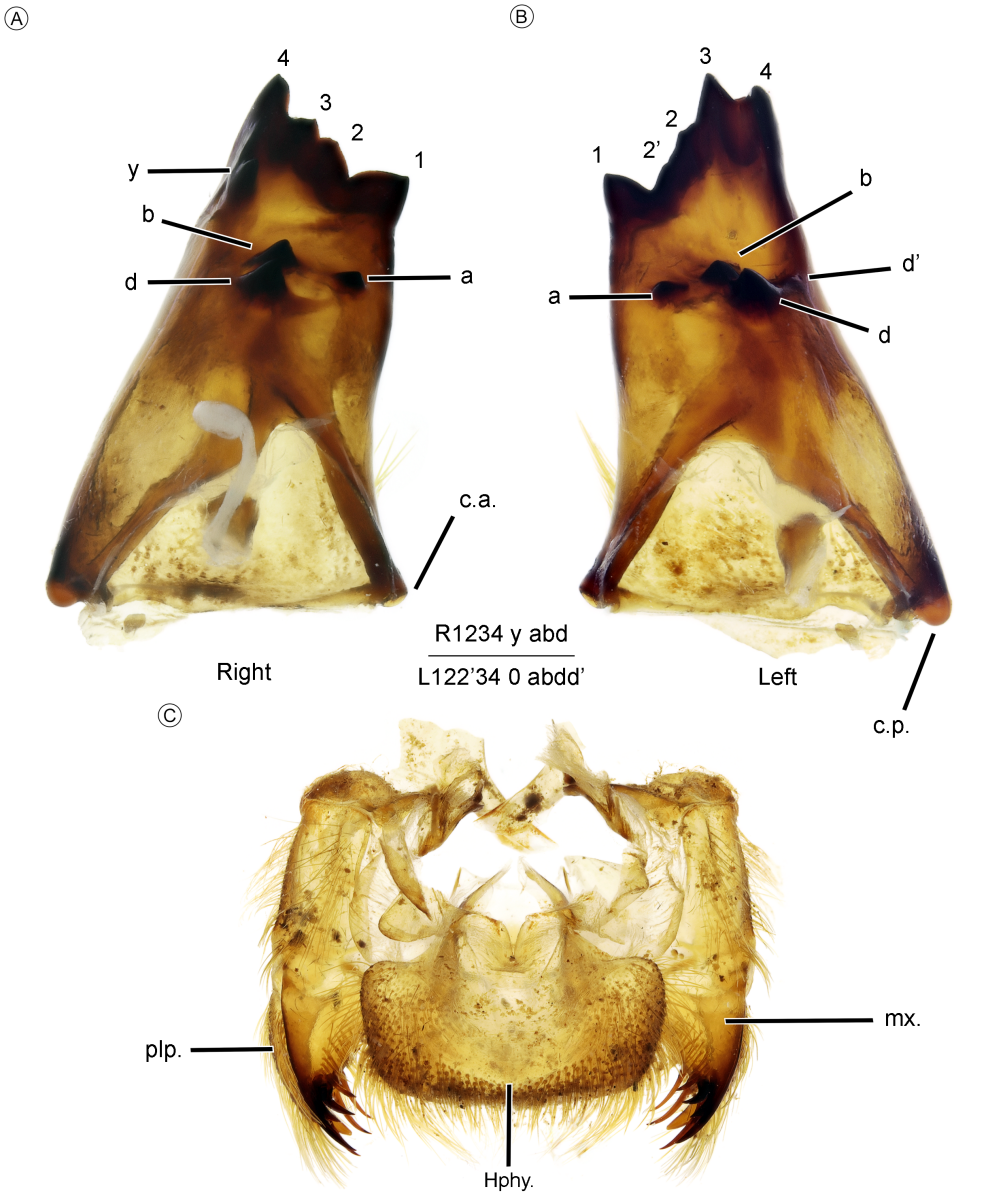
Larva mouth parts of *Neocordulia mambucabensis* from state of Paraná, Brazil (DZUP). (A–B) right and left mandibles inner surface; (C) maxilla in ventral view. Abbreviations: a. c., anterior condyle; hphy., hypopharynx; mx., maxilla; p.c., posterior condyle; plp., maxilla palp.

**Figure 9:**
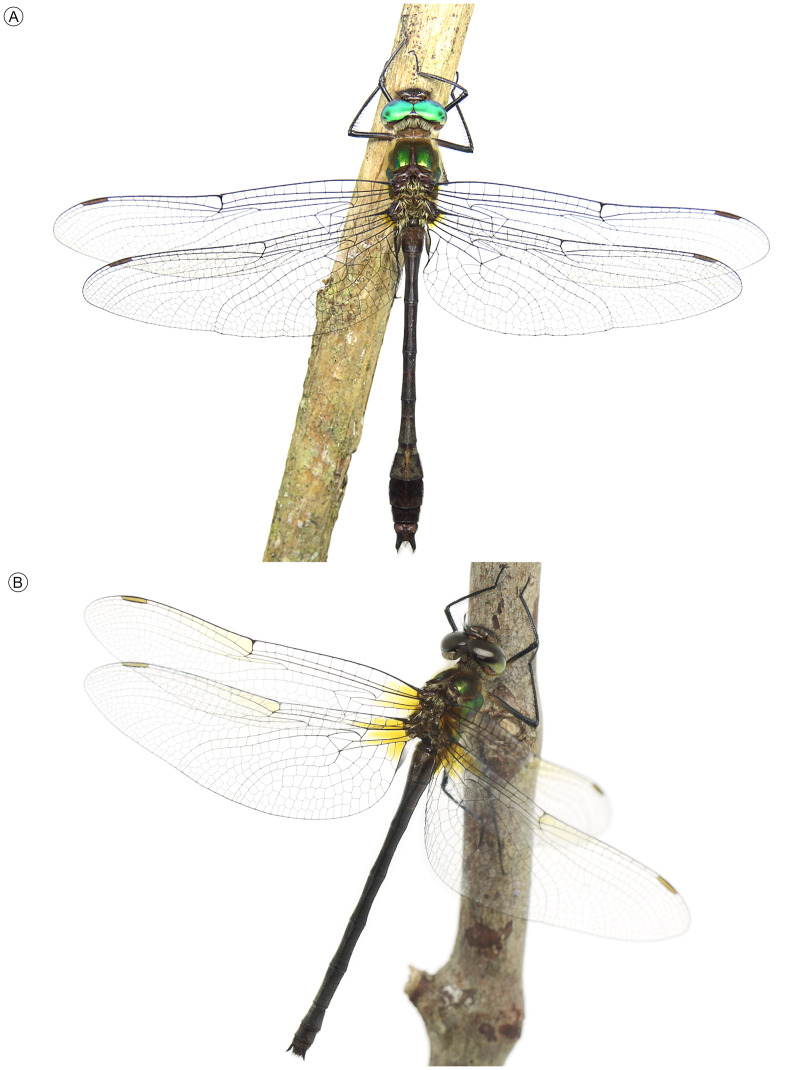
Habitus of living specimens of *Neocordulia mambucabensis* from state of Paraná, Brazil (DZUP). (A) male, (B) female. Photos: A by B. A. Araujo, B by A.P. Pinto.

*Neocordulia mambucabensis* is a well-established idomacromiid species, morphologically distinct from all other congeners, with the male, female and ultimate stadium larva now fully characterized (Figs. 1–9). Although the name-bearing type (*i.e.,* holotype) housed in MNRJ has been lost, photos of specimen habitus and collection labels have survived (Figs. 1B–1C, 1E–1F). Therefore, based on Articles 75.2 and 75.3 of the International Code of Zoological Nomenclature, there is no justification for designating a neotype, as these are not exceptional circumstances, its identity is indisputable, and it is not involved in any complex zoological problem requiring clarification of its taxonomic status. Thus, based on current knowledge, the designation of a neotype is invalid.

**Figure 10:**
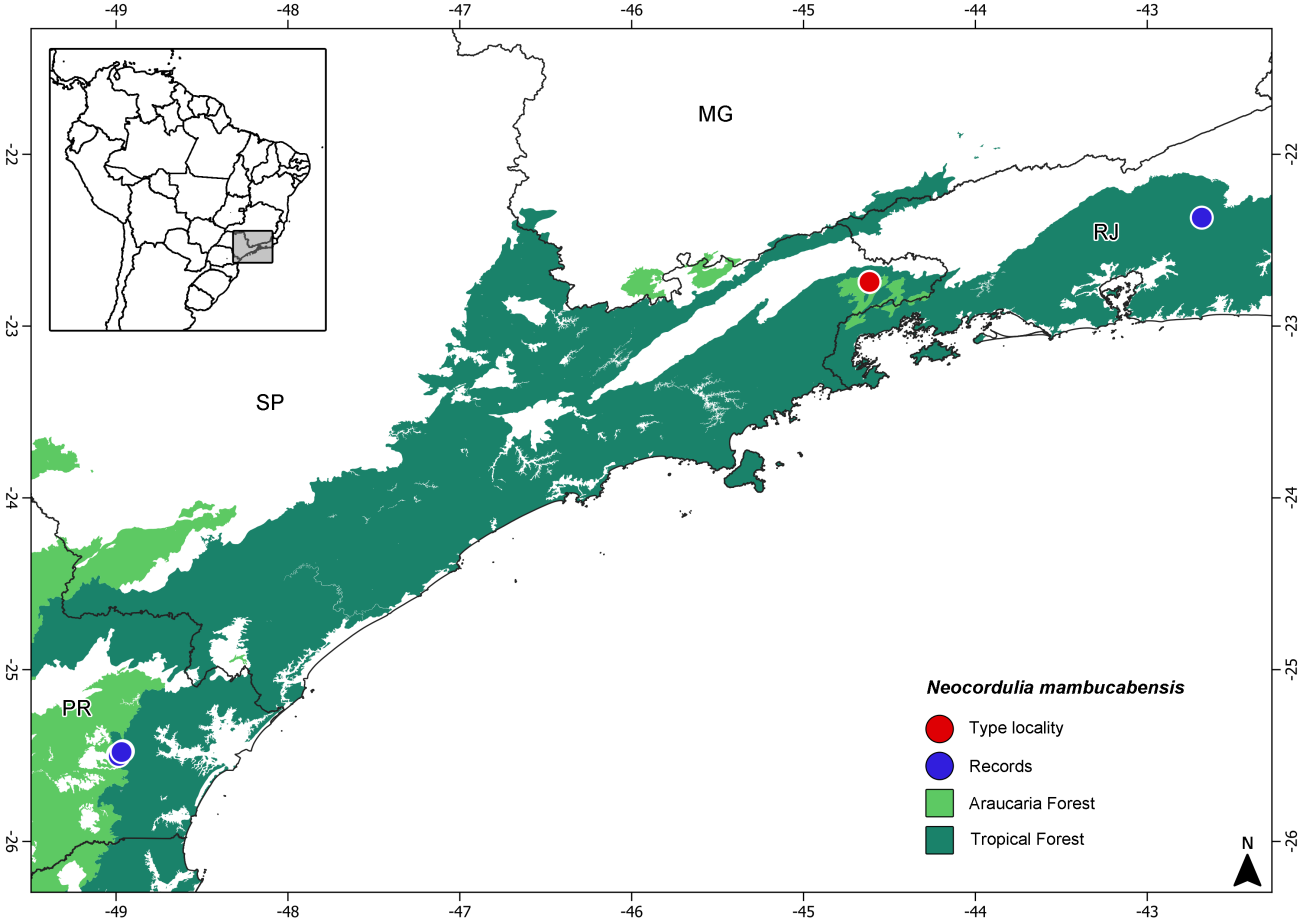
Map with distributional records of *Neocordulia mambucabensis* and vegetational type in the Atlantic Forest domain. MG, Minas Gerais State; RJ, Rio de Janeiro State; SP, São Paulo State; PR, Paraná State.

The main male diagnostic morphological characters of the *N. setifera*-like group, to which *N. mambucabensis* has been included, are compiled and compared in Table 1. Differences in coloration of costal margin, pterostigma, and femora were observed between original descriptions and holotype examination of *N. mambucabensis* (Fig. 1B). In addition, significant discrepancies were found when comparing additional specimens of *N. mambucabensis* (Figs. 2–5, 9) to its original descriptions, especially for the female (Fig. 5), in part due to a lack of information and imprecise diagnostic characters. The specimens from the type series of *N. mambucabensis* had collection labels with headquarters at Serra da Bocaina National Park, Rio Mambucaba River (Figs. 1C, 1F), hence this site was considered as the type locality (Fig. 10). Therefore, it is located in the state of São Paulo and not in Rio de Janeiro such as stated in the original description (see Discussion and also Costa & Mascarenhas, 1998).

Through the analysis of the holotype and associated specimens, we provide accurate morphological data for both male and female of *N. mambucabensis*, and their larvae are also described. A new diagnosis for the species is presented.

### Larval biology and identity

Larvae (Figs. 6–8) were collected in clear running water ecosystems, ranging from catchments of water to lotic streams and rivers. All known populations of *N. mambucabensis* occur in the Atlantic Forest domain with similar vegetation formations (Fig. 10). In the present study, we gave preference to the larvae from MASE in Paraná State (eight specimens) for larval description and diagnosis, due to poor knowledge on larval morphology and several *Neocordulia* species occurring in sympatry in the Rio Mambucaba River in São Paulo State, so additional investigation is needed to support the association of the larvae obtained herein (ten specimens) to this species, which will be addressed in a future study. The previously purported diagnostic characters for larval identification and distinction among *Neocordulia* species were not useful for our study. Most of these characters were found to represent either sexually dimorphic traits (*e.g.*, dorsal blunted processes on epiproct of male, relative size and proportions of cerci and epiproct) or individual variation (*e.g.*, number of palpal and premental setae).

### Female status and association

Based on morphological comparative analysis (Table 2), the female emerged and recently collected from the Rio Mambucaba River at Serra da Bocaina National Park (São Paulo State) agrees with the holotype, as well as the males and females from MASE, thus belonging to *Neocordulia mambucabensis*. Over almost ten years of collecting in MASE (2017–2024), we believe that the only *Neocordulia* species occurring in the visited sites is *N. mambucabensis*, as the adult males are easily associated to this species and all collected females are confidently conspecific to examined males, based on size, coloration and wing venation.

Conversely, the female described as allotype (Fig. 1E) has the wing costal margin pale, light brown (dark brown to black in the holotype Fig. 1C), legs light brown with definite contrasting coloration with the black areas on distal of femur, and articulation of femur-tibia distinct from the dark brown to black coloration, without definite contrast between pale and dark areas observed in the holotype (Fig. 1C), also in the female emerged from larvae collected in the PNSB in São Paulo and all specimens from MASE in Paraná (Figs. 1A, 1D). This paratype female (referred to as allotype in the original description) also is significantly smaller than both males and females, measuring about 45 mm of total length, differing from 55–60 mm of the other specimens, we also observed a very similar size between sexes (Table 2). The shape of lobes of the subgenital plate illustrated in Costa & Santos (2000b) does not correspond to that of any female we have studied or any other known female of the genus, looking instead to be either a distorted structure due to being young specimen reared from larva and emerged in laboratory or representing an unknown female of the genus. Taking all these issues into account, we conclude that the previously female reported as allotype does not belong to *N. mambucabensis.*

### Taxonomy

**Table:** 

***Neocordulia mambucabensis* Costa & T.C. Santos, 2000**
https://zoobank.org/NomenclaturalActs/1cfa2bb9-0512-412e-bce0-f63bcf624a00
(Figs. 1–10)

***Neocordulia*** **[(*Neocordulia*)]** ***mambucabensis*** Costa & Santos, 2000a; Costa & Santos, 2000b: 247–253, figs. 1–11 (description of holotype ♂, Brazil. Rio de Janeiro State [sic]: Serra da Bocaina, Rio Mambucaba, 22−23°S, 44−45°W, 22.XI.1979, ND Santos, SM Pereira & LF Netto leg. in MNRJ, combination implicit by the context, key, biological notes, illustrations of secondary genitalia in lateral and ventral views, S8 with sternal process in ventral view, caudal appendages in dorsal, lateral and ventral views of the holotype, S8–10 in lateral and ventral views of female paratype);—Machado (2005: 302, mention);
***Neocordulia mambucabensis*** Costa & Santos, 2000a; Costa & Santos, 2000b:—Carvalho & Kloosterman (2001: 1, mention);—Heckman (2006: 68, 69, fig. 3.2.39, key, reproduction of illustrations from Costa & Santos, 2000b);—Costa, Carrico & Santos (2010: 51, mention);—Pinto & Lamas (2010: 614, distribution);—Carriço, Costa & Santos (2011: 72, mention);—Kompier (2015: 183, 342, diagnosis, photos of habitus of male and female, caudal appendages of male, biological notes);—Araujo & Pinto (2021: 1–2, 7–8, 10–12, fig. 21, record to Paraná, taxonomic notes, photo of male habitus); —Paulson et al. (2026: catalog).
**[*Neocordulia*]** ***mambucabensis*** Costa & Santos, 2000a; Costa & Santos, 2000b:—Paulson (2004: 167, combination implicit by the context, mention);—Garrison, Von Ellenrieder & Louton (2006: 163, mention);—Garrison & Von Ellenrieder (2019: 52, mention);
**[*Neocordulia*** **(*Neocordulia*)]** ***mambucabensis*** Costa & Santos, 2000a; Costa & Santos, 2000b:—Costa & Machado (2007: 146, combination implicit by the context, mention).

**Type specimens (1♂ and 1♀).** BRAZIL. Rio de Janeiro State [sic, São Paulo]: Holotype ♂, Serra da Bocaina National Park (headquarters), Rio Mambucaba river, no. 1,388, 22.XI.1979 [larva F-0], eclosion [sic, emergence] began to 5:00 of 29.XI.[19]79, N. [D.] Santos, L. F. [Reys] Net[t]o & Sueli [M. Pereira] leg. (MNRJ); Allotype ♀ [misidentification] same data but no. 1,398, eclosion [sic, emergence] in 24.XI.[19]79 (MNRJ). Both specimens were lost in the fire of MNRJ and examined personally (APP) or by photos.

**Material examined (6♂, 6♀, 11 larvae and 10 exuviae).** BRAZIL. Rio de Janeiro State: 1♂, Nova Friburgo municipality, Fazenda Campestre, Salinas −22.369, −42.683, 1,050 m a.s.l., 07.I.2014, T. Kompier leg. (DZRJ 2435). São Paulo State: 1♀ (and its F-0 exuvia) and 1♂ F-0 exuvia, São José do Barreiro municipality, Serra da Bocaina State Park, Rio Mambucaba river −22.7431, −44.6162, 1,499 m a.s.l., sieve, 11.I.2023, A.P. Pinto & J. Ehlert leg, emerged in 02.XI.2023 (DZUP 501373, 501369, DNA LABSIA 563); 1♂ F-0 exuvia same data but 09.II.2023 (DZUP 501372); 3♂ F-0 larvae, same data but 13.II.2024, A.P. Pinto, J. Ehlert, E. Denck & R.C. Varella leg. (DZUP 501647–501649). Paraná State: 1♂ (and its F-0 exuvia), Piraquara municipality, Pico do Marumbi State Park, Mananciais da Serra Protected Area (SANEPAR), Iporan catchment −25.4800, −48.9688, 1,053 m a.s.l., riparian zone, 06.XI.2018, emerged in 17.XI.2018, B.R. Araujo leg. (DZUP 501273, DNA ODO-LABIA 126); 1 F-0 larva, same but 17.I.2018 (DZUP 501258); 1♂ (and its F-0 exuvia), same data but 07.XI.2018, emerged in 29.XI.2018 (DZUP 501271, DNA ODO-LABIA 157); 1♂ (and its F-0 exuvia), same data but Malaise trap crossing the stream, 28.XI–11.XII.2018, B.R. Araujo & A.P. Pinto leg. (DZUP 501249); 1♂, 2♀ (and its F-0 exuviae) and 1♂ F-0 larva, same data but River I −25.4956, −48.9898, 976 m a.s.l., emerged in 17.XI.2018 (male) and 03.XII.2018 (females) (DZUP 501256, 501270, 501272, 501274, DNA-LABIA 127, 136–137); 2 F-0 larvae and 3 larvae, same data but 27.X.2022 (DZUP 501267, 501578–501580); 1♀, same data but emerged 23.I.2024, J. Ehlert & A.P. Pinto leg. (DZUP 501371, DNA LABSIA 617); 1♀ (and its F-0 exuvia), same data but 09.XI.2022, J. Ehlert leg. (DZUP 503175, DNA LABSIA 425); 1♂ larva and 1♂ F-0 exuvia, same data but −25.4964, −48.9817, 1,021 m a.s.l., A.P. Pinto, B.R. Araujo & A.C. Domahovski leg. (DZUP 501255, 501650); 1♂ and 1 exuvia, same data but Salto catchment −25.5028, −48.9853, 1,030 m a.s.l., 17.I.2018, B.R. Araujo leg. (DZUP 500547, 501258, DNA LABIA 204); 1♀, same but data Ipiranguinha catchment −25.475, −48.9612, 1,074 m a.s.l., Malaise trap crossing the stream, 17.XI.2023–07.XII.2023, A.P. Pinto, E. Denck, L. Polizeli & R.C. Varella leg. (DZUP 501608).

**Table 1:** Comparison of morphological characters. Morphological diagnostic characters of males of *Neocordulia setifera*-group, L, literature data and O, observed data. Observed data on name-bearing type of *N. machadoi* obtained by photos. Literature data for *N. machadoi* from Santos, Costa & Carriço (2010); for *N. mambucabensis* from Costa & Santos (2000b); for *N. matutuensis* from Machado (2005); for *N. setifera* and *N. volxemi* from May (1992).

**Character/species**		***N. machadoi*** **(holotype)**	***N. mambucabensis*** **(incl. holotype)**	***N. matutuensis*** **(incl. holotype)**	***N. setifera*** **(incl. lectotype)**	***N. volxemi*** **( - )**
1. Costal margin	L	Black	Black	Black	Dark brown, black	Pale yellow brown
	O	Light brown	Dark brown	Dark brown	Dark brown	Pale yellowish-brown
2. Pterostigma	L	Light brown	Light brown	Dark brown	Brown	Pale yellow brown
	O	Light brown	Brown	Dark brown	Dark brown	Yellowish brown
3. Femur coloration	L	Black	Light brown	Brown with $ \frac{1}{3} $ black	Dark brown, black	Orange brown, shading to dark brown at apexes
	O	Brown with $ \frac{1}{3} $ black	Brown with $ \frac{2}{3} $ black	Brown with $ \frac{1}{3} $ black	Dark brown with $ \frac{1}{3} $ black	Light brown with $ \frac{1}{3} $ brown
4. Genital lobe	L	Quadrangular	Quadrangular	Rounded	Blunt at tip, slightly excavated posterodistally	Blunt apically
	O	Quadrangular	Quadrangular, apex slightly projected	Rounded tip, projected at base	Quadrangular, rounded pronounced tip with excavation	Quadrangular, apex slightly projected
5. Sternal process on S8	L	Small conical	Prominent biconical	Conic	Biconical	Prominent biconical
	O	Rounded, circular	Prominent biconical	Conic	Biconical	Biconical
6. Cercus lateral margin in dorsal view	L	–	–	A small basal tubercle and another at 3/4 length	Angled inward and convergent in distal 1/4	Tooth at about 3/4 length
	O	Small projection at about 3/4	Straight, smooth	Small basal tubercle and another at 3/4	Angled projection at 3/4 leading to a convergent apex	Small projection at about 3/4
7. Cercus inner margin	L	–	Small protuberance in basal half	A very large mediobasal projection	upturned medial tooth at 1/3 length	Blunt medial tooth at about 2/5 length
	O	Small projection at about 1/3 basally	Angled projection at basal half, apex at center of cercus	Strong angled projection mesially, touching apexes	Strong angled projection mesially, touching apexes	Small projection at about 1/3 basally
8. Epiproct shape	L	Distal margin slightly angulate medially with two teeth	Broad at base and slightly narrowed at about half length, convergent in distal half		Lateral margins moderately convex in basal 1/2, then strongly constricted, apex bifid	Lateral margins moderately convex in basal 1/2, concave in distal 1/2, apex emarginate
	O	–	Quadrated	Triangular	Triangular	Triangular

**Table 2:** Morphological comparison between type series and additional females of *Neocordulia mambucabensis*.

**Character/species**	**Holotype male**	**“Allotype”**	**Females from MASE** **(*N* = 5)**	**Topotype female from PNSB**
	**Type specimens**		**Additional specimens**	
1. Total length	54 mm	45 mm	60 mm^*^	55 mm
2. Costal margin coloration	Dark brown	Light brown	Dark brown	Dark brown
3. Pt coloration	Light brown	Pale yellow	Light brown to brown	Light brown
4. Number of Ax in FW	12	10	12	12
5. Fw total length	39 mm	35 mm	39–41 mm	–
6. Femur coloration	Brown gradually darkening to black at distal portion	Light brown with distal 1/3 dark brown	Brown gradually darkening to black at apex	Light brown with distal 1/3 dark brown
7. Tibia coloration	Dark brown	Light brown	Dark brown	Dark brown
8. S9–10 length	3 mm	2 mm	3 mm^*^	2.6 mm
9. subgenital plate length	–	0.3 mm	0.25 mm^*^	–

**Notes.**

MASE Mananciais da Serra Protected Area, Paraná State PNSB Serra da Bocaina National Park, São Paulo State

* only well-preserved female measured.

### Diagnosis

Adult (Figs. 1–5, 9). Medium to large-sized emerald dragonfly, body ground color predominantly brown to dark brown, thorax with bright green metallic reflections and dense hair-like pilosity (Fig. 9). Both sexes of *N. mambucabensis* can be distinguished from all species of *Mesocordulia* and *Neocordulia* s.s. by its large size Fw 40–41; labrum and clypeus pale, orange-brown; costal margin dark (Fig. 2); metathoracic femur long (8.40–8.96), dark colored (Figs. 1A, 1D, 9C), with at least 0.5 distal dark brown to black in paler specimens; cercus dark (Figs. 4D–4F, 5, 9).

The combination of following characters will allow one to distinguish males of *N. mambucabensis* (Fig. 9A) from all other species within *Neocordulia* s.l.: costal margin dark; legs largely dark, pale on 0.3–0.5 basal of femur and entire trochanter and coxa; genital lobe roughly quadrangular (Fig. 3A), ventral edge straight produced ventrally into a thumb-like tip, covered with stiff dark seta anteriorly; posterior hamule slightly higher than genital lobe with external branch long and stout, and inner branch not visible in lateral view, inner branch with five denticles in ventral view (Fig. 3B); sternal process of S8 biconical, with a pair of large and slightly divergent thorns-like tubercles (Figs. 4A–4C); epiproct (Figs. 4D–4F) pale, quadrangular, wide at full length, ratio of maximum width/width at tip ≥ 0.95; cercus (Figs. 4D–4F) robust, dark, flattened, tips rounded, lateral margin almost straight, inner margin convex with an single rounded angulation at 0.33 basal and covered with dense black hair-like setae, a large black ventrobasal tooth, strongly projected outward.

Females of *Neocordulia* s.l. are poorly known and badly characterized. In addition, they are very similar morphologically, which hampers efforts for clear-cut diagnoses. However, we provide a tentative diagnosis here. Long legs (metathoracic femur ≥ 8.0 mm) distinguish *N. mambucabensis* from all *Mesocordulia* species (metathoracic femur ≤ 6.6 mm). A dark brown costal margin and cercus (Figs. 2B, 5, 9) help to distinguish *N. mambucabensis* from *N. androgynis* and *N. volxemi* which has a pale yellow costal margin and cercus. This may also be true for all species with pale costal margins whose females are still unknown, i.e.: *N. fiorentini*, *N. gaucha*, *N. machadoi*, and *N. santacatarinensis*. From the females of *Neocordulia* s.s. species which have a dark costal margin, the subgenital plate (Fig. 5B) with S8 sternite projected into a triangular convex basal process and large subgenital lobes ≥ 0.4 of total S9 length, forming a pair of triangular blades help to distinguishing *N. mambucabensis* from all these species (base of subgenital plate straight and not projected in *N. biancoi*; lobes shorter in *N. setifera;* subgenital lobes length ≤ 0.3 of total S9 length in all of them)*.* The females of *N. pedroi* and *N. matutuensis* are still unknown but we assume they would be strongly similar to *N. setifera* and most likely the same characters would allow its distinction from those species, while for *N. maurocostai*, also known only from males, distinction should be based on the distinct geographic occurrence (Amazonia *vs.* Atlantic Forest) and association with males.

*Neocordulia mambucabensis* is a very distinctive species, thus it can be only misidentified with potentially sympatric Atlantic Forest large species, *i.e., N. matutuensis* and *N. setifera.* It is unique based on the large size (total length 57–60), dark costal margin, long legs (metafemur 8.40–8.96), abdomen almost entirely dark brown to black (Figs. 1A–1B, 1C–1D, 9), lacking pale spots, hamule bifurcated into a smaller external and a reduced inner branches (Fig. 3B), cercus dark brown to black (Figs. 4D–4F), with inner margin angulated and tip with a tuft of setae. Males can be separated from these two species by the biconical S8 sternal process (Figs. 4A–4C*;* conical in *N. matutuensis*), epiproct largely quadrangular (Fig. 4E), flattened cercus (Figs. 4D–4E) with inner margin gently convex, external margin straight in dorsal view, lacking processes (epiproct triangular, cercus inflated, cercus with inner margin strongly projected into a large and stout tubercle, external margin sinuous, bearing a medio- and apical tubercle-like processes in *N. matutuensis* and *N. setifera*). Females of these species are virtually undistinguishable, except by the already mentioned characteristics.

Larvae (Figs. 6–8). Large sized larvae, body color predominantly brown (Figs. 6A–6B), covered with short scale-like setae, legs long, posterior longer than abdomen length. The combination of the following characters allows one to distinguish *N. mambucabensis* from all *Mesocordulia* larvae (character given in parenthesis): Frontal plate (Fig. 6C) anterior margin convex (slightly truncated, with shallow furrow in some species) and reaching maximum 0.5 of antenna length (at least 0.9 of antenna); occipital angle slightly rounded (Fig. 6D, sinuate, projected into distinct lobe); 11–12 premental setae (Fig. 7A; 10 or less), seven palpal setae (Fig. 7E; ≤ 6); male cercus tip convergent (Fig. 6E; divergent).

For *Neocordulia* s.s., larvae are very similar, and the ultimate stadium (F-0) was previously described for only a few species. They are virtually undistinguishable except for small differences in the number of premental setae and palpal crenulations (Fig. 7); however, these can vary among specimens and sometimes vary along the bilateral axes of the same individual. Currently, *N. mambucabensis* can putatively be distinguished from most species by F-0 total length of 25–27 mm, except for *N. volxemi* (27 mm) and *N. pedroi* (26 mm).

### Additions to the adult male morphology

*Secondary genitalia* (Fig. 3). Anterior lamina pale, edges darker, posterior margin—composed by anterior lamina and anterior hamule—triconcave, bearing golden hair-like setae on the edges of central concavity. Posterior hamule robust, dark brown, apex bifurcated into external and inner branches, higher than genital lobe in lateral view; external branch with a distal rounded projection, tip slightly acute in lateral view; inner branch with a row of five denticles at inner margin. Genital lobe well-developed, brown with golden pilosity, quadrangular; slightly oblique and reaching 0.3 of posterior field of S2; inner margin projected upwards, bearing dense dark setae distally; external margin slightly excavated. *Vesica spermalis*. V1 with ventral surface with a small acute pair of processes at base, covered with short golden setae; V2 cylindrical and smooth; V4 with one flagellum.

*Abdomen*. Auricles light brown, rounded, tip shallowly projected. S8–10 (Fig. 4) ventrally with pale areas, sternite light brown; S8 with a large biconical sternal process (Figs. 4A–4C), projected caudally into a pair of small, acute and slightly divergent thorns, light brown basally, darkening to brown apically. A dense complex of dark setae on posterior portion of S8 sternite (Fig. 4B); dorsal surface of S10 with a small protuberance, not forming a definite keel (Fig. 4F). Cercus dark brown (Figs. 4D–4F), divergent tips, cylindrical at base and flattened dorsoventrally at posterior 0.7, with large ventrobasal tooth projected outward laterally; in dorsal view external margin slightly rounded and smooth; inner margin with basal projection at 0.4, than straight until apex, covered by long dark setae; apex rounded and almost parallel; in lateral view reaching 0.8 of S9 + 10. Epiproct large (Fig. 4E), slightly bifurcated as a V-shaped at apical margin, reaching 0.7 of cercus length; light brown, darkening at margins, with dark brown acute spine at apex laterally projected.

### Description of adult female

*Head* (Fig. 1D). Labium and labrum yellowish-brown to dark brown. Clypeus orange-brown. Antefrons dark brown on superior margin slightly fading to brown at base; postfrons dark brown with a deep central furrow. Antenna dark brown. Vertex brown, covered with long dark hair-like setae. Occiput convex, triangle brown, dorsally almost twice as long as eye seam.

*Thorax* (Fig. 1D). Prothorax yellowish-brown. Synthorax ground color light brown to brown with blue and green metallic reflections, covered with dense long whitish-golden setae; antealar process brown with no metallic reflections. Coxae and trochanter largely yellowish-brown, with few darker areas. Femur light brown, darkening to dark brown covering 0.5–0.7; tibia and tarsus dark brown.

*Wings* (Fig. 2B). Membrane hyaline, a basal yellow spot at level of arc distally and a yellow faded spot at nodus; costal margin and venation dark brown; Ax: 11–14 in Fw, 8 in Hw; Px: 8–12 in Fw, 9–15 in Hw; arc at 2nd Ax in all wings; sectors of arc stalked; two bridge crossveins in all wings (a single Fw has one bridge crossvein see Fig. 2B); discoidal triangles, subtriangles and supratriangles free in all wings; discoidal field with two cells rows almost until wing margin, expanding to four or five cells at distal end. Cu-A space with one crossvein in Fw and two in Hw. Anal loop elongated, two cells row with well-defined midrib (Cuspl) and no sole, totaling 17 cells.

*Abdomen* (Figs. 1D, 5, 9B). Cylindrical, predominantly brown shading to dark brown (Figs. 1D, 9B); S1–3 yellowish-brown, S3 darkening to dark brown on posterior half, S4–10 dark brown; S7–8 ventral surface with yellow areas; dorsal carinae in all segments; without lateral carina in all segments; S2–8 ventral carina pale, yellowish-brown; transverse carina (TC) dark brown, medially on S2–5 and anteriorly on S6–7, lighter in S2; Posterior field brown to dark brown. Sternites dark brown, lightning to brown in S7–10. Caudal appendages short (Fig. 5); cercus dark brown to black, as long as 0.5 of S9+10, cylindrical at base, very acute apically, covered with dark setae, divergent apex. Epiproct dark (Fig. 5) brown, covered with long dark setae, as long as 0.66 of cercus.

*External genitalia* (Fig. 5). Subgenital plate dark brown, as long as 0.4 of S9 total length, divided in two lobes; posterior margin of sternite of S8 (base of subgenital plate) mesially projected into a triangular acute process; subgenital lobes (Fig. 5B) dark brown to black, triangular, with acute Y- or V-shaped deep cleft, external margin slightly convex, bearing long brown hair-like setae on distal half; pair of small rudiments of valvae.

### Description of ultimate (F-0) larval stadium

*Head* (Figs. 6A–6D)*.* Wider than longer, roughly pentagonal in dorsal view, occipital angle gently rounded (Fig. 6D), posterior margin slightly concave, cephalic capsule somewhat flattened dorsoventrally, brown, patterned with dark-brown spots, bands and stripes, covered with short scale-like and spatulated setae. Frons (Fig. 6C) pale brown with small triangular dark-brown spot posteromesally, protruded into a large, rounded plate, anterior margin covered by short scale-like setae. Vertex dark brown, this spot tapering laterally up to the level of midlength of eyes, pale on ocelli, covered by short scale-like setae. Epicranium brown with dark brown marks on setose areas, a pair of rounded spots posterior to eyes and outlined by a dark brown stripe, dorsal surface covered by a distinctly pattern of scale-like setae surrounding glabrous areas (muscular impressions sensu Carvalho & Kloosterman, 2001), narrow transverse rows of setae posteriorly of eyes, a pair of distinct tuft of scale-like setae close to coronal suture, occipital angle (Fig. 6D) dorsally covered by short scale-like and lateroventrally by long spatulate and spiniform setae. Functional area of compound eye about of 0.4 of total eye area, elevated dorsally into a rounded conical prominence; Antennifer, scape, articulation of pedicel and half of first flagellomere dark brown (Fig. 6C), membrane and rest of antenna whitish-yellow; antenna cylindrical, 7-segmented, flagellomere length 4 >1 >5 >3 >2 , acute at apex; scape and pedicel covered by scale-like setae laterally.

*Mouth parts* (Figs. 7 and 8). Labium deeply scooped elongated ladle-shaped, typical of most Cavilabiata; lateral margin about of basal 0.3 of prementum concave (Figs. 7A–7B) with a long row of short hair-like setae, similar to the premental setae; ligula (median lobe) entire, without cleft, projected into triangular distal margin bearing small spiniform setae; two sets of premental setae with 12–14 (Fig. 7A). Labial palp triangular, distal margin with nine crenulations (eight but first bifurcated) bearing 14–19 (Figs. 7A–7B, 7E) strong setae at apex; outer and inner margin bearing spiniform setae, the last longer distally and gradually decreasing proximally; seven palpal setae, base with 24–29 spiniform setae and one setella (Fig. 7E); movable hook at least 3 times longer than first crenulation (Figs. 7C–7E). Mandibular formula (Figs. 8A–8B) R 1234 y abd, L 122′34 0 abdd’; maxillar lacinia (Fig. 8C) with eight elongated and strong teeth.

*Thorax* (Figs. 6A–6B). Prothorax anterior margin with long hair-like setae; pronotum rectangular, all margins covered by small scale-like setae (Fig. 6A); lateral margin projected, posterior margin sinuous, with a pair of distal concavities occupying 0.4 on each side. Legs (Figs. 6A–6B) longer than abdomen length; femur dark brown with yellow spots, tibia yellowish-brown with a dark spot basally, both covered with long spurs; tarsus 3-segmented, with short spines ventrally, pretarsal claws long and pale yellow. Wing sheaths (Fig. 6A) reaching posteriorly S5, posterior margins with double rows of setae; ventral thoracic labial concavity surrounded by scale like setae.

*Abdomen* (Figs. 6A–6B)*.* Longer than wide, maximum width at S5–6; light yellowish-brown at S1–4, dark brown at S8–10; dorsal surface with four large pale spots at S5–8; all segments lateral margin bearing small setae, S8–9 with small lateral spines (Figs. 6A–6B); no dorsal spines, but a shallow tuft of small setae at dorsal spine area on S3–9; posterior field of S5–9 with dorsal small rounded white spots; S9 posterior margin concave (Figs. 6E–6F); male anal appendages (Fig. 6E) triangular, acute tip; cercus apex convergent; epiproct enlarged basally, dorsally with a tubercule like processes at middle; paraproct long, covered by dense setae at inner margin.

**Measurements (mm). Adult male (*n* = 4):** Total length 57–60; abdomen length (without caudal appendages) 39–42; head maximum width 8.0; eye seam length 0.68–0.78; Fw length 40–41; Fw maximum width 8–9; Hw length 40.5–41.0; Hw maximum width 12.0–12.5; pt length in Fw 2.84–3.12, in Hw 2.93–3.12; length of metathoracic femur 8.40–8.96; length of metathoracic tibiae 9.12–9.52; metathoracic tibial keel 4.0; mesotibial keel proportion 0.35–0.40; S9–10 length 3.28–3.59; cercus length 2.68–2.90; epiproct length 2.0–2.1. **Adult female (*n* = 4):** Total length 55–60; abdomen length (without caudal appendages) 36; head maximum width 8.0–8.8; eye seam length 0.84–0.87; Fw maximum width 9.5; Fw length 39–41; Hw maximum width 13.6–14.3; Hw length 40; pt length in Fw 3.00–3.08, in Hw 3.15–3.21; length of metathoracic femur 8.56–8.80; S9 length 1.62–1,72; S9–10 length 2.37–2.90; subgenital plate length 2.75–2.90; cercus length 1.35–1.44. **Larvae F-0 (*n* = 5):** Total length 25–27; head length 3.87–4.25; maximum width of head 6.87–7.18; wing sheaths length 7.18–7.50; maximum width of abdomen (S5–6) 8.4–8.8; abdomen length with appendages 16.2; S8 lateral spines length 0.32–0.40; S9 lateral spines length 0.4–0.6; profemur 5.38; mesofemur 7.36–7.60; metafemur 8.4–8.8; protibia 6.4–6.9; mesotibia 8.16–8.50; metatibia 8.90–9.36.

### Remarks

Both the holotype of *N*. *mambucabensis* and the paratype female (allotype) were described based on reared larvae that emerged in a laboratory. This crucial information was omitted in the original description, but through review of images of the specimens and by reviewing collecting data from the paper envelopes (Figs. 1B–1C, 1E–1F) it was possible to obtain the emergence date for both male (November 29th, 1979) and female (November 24th, 1979). Differences in the coloration of emerged and mature adults collected on the field were observed. Some of the emerged specimens presented a darker general coloration, most likely due to postmortem changes and/or bad preservation, except for the pterostigma, which is lighter than mature specimens. In Costa & Santos (2000b), the femur and tibia of males were described as light brown, which was a unique characteristic of *N. carlochagasi*. However, after analyzing holotype photos (Figs. 1B, 1E) and additional material of both males and females of *N. mambucabensis* (Figs. 1A, 1D, 9), except for the female allotype (Fig. 1E), all specimens present a dark brown femur (lighter at base) and tibia.

### Biological data

Adults and larvae of *Neocordulia mambucabensis* dragonflies inhabit forested areas, located at high elevation at least about 1,000 m a.s.l. All records are from the Brazilian Atlantic Forest domain, with collecting sites ranging from 976 m a.s.l. at 25° S of latitude in Paraná State to 1,499 m a.s.l. at 22° S of latitude in Mambucaba River, São Paulo State (Fig. 10). All larvae were collected in clean, crystal water at small streams and rivers, usually in riparian zones at the margin of water bodies, sometimes at the bottom of leaf litter. One adult female was collected using a Malaise trap, positioned crossing a stream that was dammed as part of an old water supply reservoir. The ultimate larval stadium specimens were mainly collected from October to January, and emergence occurred overnight (probably in the early morning hours), from September to November.

## Discussion

Libelluloidea represents a species rich, ubiquitous and key group of dragonflies, being the most abundant and widespread anisopteran group. Recent studies have supported its monophyly (see Ware, May & Kjer, 2007; Dijkstra et al., 2013; Carle, Kjer & May, 2015; Bybee et al., 2021; Goodman et al., 2026; Newton et al., 2026), but there are still many evolutionary, phylogenetic and taxonomic issues pending further investigation in the superfamily genera, since the species relationships are particularly in need of revision and have been intriguing odonatologists for decades. *Neocordulia* s.l. has two centers of diversity, one in tropical South America, especially in a hotspot for conservation (the Atlantic Forest) for *Neocordulia* s.s. and a second in Northwest South America to Central America for *Mesocordulia.* The putatively close phylogenetic relationships of these taxa with African, European and Australasian genera of Idomacromiidae suggests an ancient diversification during an early configuration of the supercontinent Gondwana (*e.g.*, Goodman et al., 2026; Newton et al., 2026). Clear-cut diagnoses for both generic and species-levels are the very first steps to start to disentangle many key issues on *Neocordulia* complex.

Although placed in the *Neocordulia setifera*-group by Costa & Santos (2000b), *N. mambucabensis* also possesses characteristics from another group in the genus—*Neocordulia androgynis*-group. The quadrate epiproct in ventral view and cercus shape in lateral view of the male are shared with *N. androgynis* and *N. carlochagasi,* while its large body size and cercus with angulations or projections in male is like *N. setifera* and *N. matutuensis*, members of the *Neocordulia setifera*-group. In addition, *N. mambucabensis* has ventral pale areas on the abdomen, which can be found in most species of the *N. androgynis*-like group, but also in *N. volxemi* and *N. machadoi*, from the *N. setifera*-group. As currently arranged, these putative groups do not reflect the phylogenetic history among species of *Neocordulia*, especially since its limits, at both subgenera and species level, are strongly supported by male characteristics only. Based on the current knowledge, these group of species first proposed by May (1992) and modified with the descriptions of new species for further authors are no longer supported, even if they were erected as no more than a tentatively arbitrary arrangement. These hypotheses should be investigated under the framework of a phylogenetic analysis.

Female identification and characterization present a significant challenge, resulting in several taxonomic barriers as misidentifications. Even though all species of *Mesocordulia* have descriptions of females, for *Neocordulia* s.s. species, only half of them are known, which leads to confusion about specimens’ associations. These difficulties are illustrated when considering the female described as an “allotype” of *N. mambucabensis*. This specimen had been tentatively identified many times independently by Newton D. Santos and Janira M. Costa, first as *N. setifera*, then as *N. androgynis* and finally as *N. carlochagasi*, the last ones with a question mark between parentheses (Fig. 1F). That specimen is now lost so further examination is no longer possible, but due to exam of its digital specimen (Fig. 1D) and its described leg coloration and body size most likely it represents a female of *N. carlochagasi*. Further supporting this hypothesis, both *N. mambucabensis* and *N. carlochagasi* are highland species, based on the elevation of the sites where these two species were recorded (Santos, 1967; Costa et al., 2000; Carvalho & Kloosterman, 2001, and this study). In addition, specimens of these two species were collected at the same sites above 1,000 m a.s.l. in the highlands of Serra dos Órgãos (Kompier, 2015). The female illustrated as *N. mambucabensis* by Kompier (2015) presents same morphology as the allotype specimen (Fig. 1E). Finally, the type locality provided for *N. mambucabensis* has a dubious location and may have been mistakenly cited as from the Rio de Janeiro State in the original description. The larvae cited in Costa & Santos (2000b) were most likely collected at highlands of the headquarters of the Serra da Bocaina National Park at São José do Barreiro municipality, state of São Paulo (IBAMA, 2002; Figs. 1C–1F, original labels), because all other known specimens were collected in high elevation sites (see Kompier, 2015; Araujo & Pinto, 2021) and the headquarters in Rio de Janeiro is located at sea level. This is corroborated by Costa & Mascarenhas (1998: 6, 7, 10, 11), who reported that the Bocaina material collected during field expeditions conducted by N. D. Santos belongs to São Paulo State.

The morphological and taxonomic knowledge of *Neocordulia* larvae are far less advanced, and any difficulties mentioned in relation to adults extend to the immature’s stages of *Neocordulia* s.l. There are few larvae described, and most descriptions rely on poor diagnostic characters and small numbers of examined specimens. Although the larval stage can be very effective for distinction at the subgenera level, as already mentioned by Pinto & Carvalho (2011) when discussing the questionable placement of *N. pedroi* in *Mesocordulia*, the distinction between species is currently unsuitable. Unfortunately, since the 2000′s—except for species such as *N. batesi longipollex*, *N. biancoi* and *N. carlochagasi* (De Marmels, 1990; Novelo-Gutierrez & Ramírez, 1995; Carvalho & Kloosterman, 2001)—larval descriptions have been plagued by misidentifications, inadequate illustrations, and incomplete treatments laiden with inaccuracies in key characters (*e.g.*, number of palpal setae). These limitations also apply to currently available keys, since they rely on sexual characteristics for the couplets (Costa & Santos, 2000a; Heckman, 2006; Datto-Liberato, Cezário & Guillermo-Ferreira, 2022). Additionally, the most recent key (Datto-Liberato, Cezário & Guillermo-Ferreira, 2022) also omits the larvae of *N. caudacuta* (De Marmels, 2008) and *N. carlochagasi* (Carvalho & Kloosterman, 2001). Therefore, based on the available descriptions and considering larval morphology alone, *Neocordulia* species are virtually indistinct.

The difficulty in recognizing different *Neocordulia* species and evaluating which names are valid, as well as its phylogenetic position relative to other corduliid genera, is reflected by the numerous inconsistences, mistakes and imprecisions associated to the species-level taxonomy in this genus. These limitations affect the reliability of information, ranging from wrong distribution records (see Pinto & Carvalho, 2011) to misinterpretation of morphological characters (Table 1, holotype divergences) and are aggravated by the scarcity of data and available specimens of corduliids in collections (*cf.* Pinto & Carvalho, 2010). Contemporary taxonomy approaches can help solve these bottlenecks with comprehensive and comparative studies, providing solid species diagnoses based on multiple sources of evidence into an integrative investigation framework (*e.g.*, new characters, all life stages, behavior and phylogenetic analysis using molecular data; Pinto, Bota-Sierra & Marinov, 2023).

## Conclusions

Males, females and larvae of *Neocordulia mambucabensis* are now characterized, illustrated and diagnosed, with updated records of occurrence (Fig. 10). The female allotype previously described for *N. mambucabensis* do not correspond to this species. Immatures are pending profound morphological and taxonomic investigation. The numerous inaccuracies in the descriptions and illustrations, along with other aspects previously discussed about *Neocordulia* taxonomy, support the necessity of a new revision, since the last one provided by May (1992) became obsolete. Based on the information currently available, it is not possible to securely provide adequate diagnoses for all *Neocordulia* species. Designating a neotype for *Neocordulia mambucabensis* is undesirable, given current taxonomic assumptions and the provisions of the International Code of Zoological Nomenclature; such a designation would constitute an invalid nomenclatural act.

### Additional material examined

*Neocordulia matutuensis* (3♂). BRAZIL. Minas Gerais State: holotype ♂, Aiuruoca municipality, Matutu Valley [−21.976, −44.603], 30.XI.1999, P.A. Machado & M. Franca leg. (ABMM). Rio de Janeiro State: 2♂, Itatiaia municipality, PNI [Itatiaia National Park], Complexo do Maromba, Cachoeira Véu-da-Noiva, Coleta-04, PNI-M2A (22°25′36.10″S, 44°37′05.80″W [−22.3300, −44.6150], 1,153 m a.s.l.), 2.X–2.XI.2015, BIOTA FAPERJ leg. (DZUP 501275, 501276).
*Neocordulia setifera* (3♂). BRAZIL. Rio de Janeiro State: lectotype ♂ (RBINS); 2♂, Itatiaia municipality, Itatiaia National Park, Setor Lago Azul (−22.4503, −44.6153, 830 m a.s.l.), 14.I.2023, A.P. Pinto & J. Ehlert leg., emerged in laboratory (DZUP 501366, 501367).
*Neocordulia volxemi* (4♂). BRAZIL. Distrito Federal: 1♂, Brasília municipality, Reserva Ecológica do IBGE (RECOR) −15.9500, −47.8818, 1,134 m a.s.l., 30.IX.2023, A.P. Pinto leg. (DZUP 501370); Minas Gerais State: 1♂, Itamonte municipality, Cachoeira do Escorrega [−22.2202, −44.8211], 17–23.IX.2011, Mascarenhas & Pereira leg. (MNRJ); 1♂, Tiradentes municipality [−21.1100, −44.1778], 02.II.2011, Lucio Bedê leg. (ABMM); 1♂, same but 14.XII.2011 (ABMM).

## Additional Information and Declarations

### Field Study Permissions

The following information was supplied relating to field study approvals (*i.e.*, approving body and any reference numbers): Collecting permits were provided by Instituto Chico Mendes de Conservação da Biodiversidade–ICMBIO/SISBIO (#59681) and Instituto Água e Terra–IAT (04.18, 037.2018, 03.20 and 52 _23).

### Data Availability

The following information was supplied regarding data availability: The raw data is available in the Material & Methods and Results.
